# Supplementary material for: SYN-View: A Phylogeny-Based Synteny Exploration Tool for the Identification of Gene Clusters Linked to Antibiotic Resistance
Source: Molecules. 2020 Dec 31;26(1):144. doi: 10.3390/molecules26010144 (PMC7795190; doi:10.3390/molecules26010144)
Supplement: Supplementary file 1 [file molecules-26-00144-s001.pdf]

Supplementary Materials: SYN-view, a Phylogeny Based Synteny Exploration Tool for the Identification of Gene Clusters Linked to Antibiotic Resistance

Jason Stahlecker[1], Erik Mingyar[1], Nadine Ziemert[1,2], Mehmet Direnç Mungan[1,2]*

1. Contents of the Result Folder

In the results directory (default is the current directory) the "*.faa" file of the corresponding "*.gbk/gbff" file is created. Additionally, a folder named "SYN-view_results" is created in which all other files are saved. Most importantly the subfolder "RESULTS" contains all results as *svg* and *txt* files. The folder "protein_faa" contains all ".faa" files of either the genome_gbff folder (default) or the specified genomes. For each hit in the input.gbk a query folder is created. In hmm mode all hmm runs are saved in the folder "SYN-view_results", while in protein mode the blast databases of the genomes of the relatives are saved in the folder "blast_databases" and of the query in "SYN-view_results". Moreover, temporary files, needed for transfer of information are saved in "SYN-view_results". The query folders contain the blast results and all svg files. The supplementary folder only contains the input parameters and the "RESULTS" subfolder.

2. Figures of the Results of Table 1

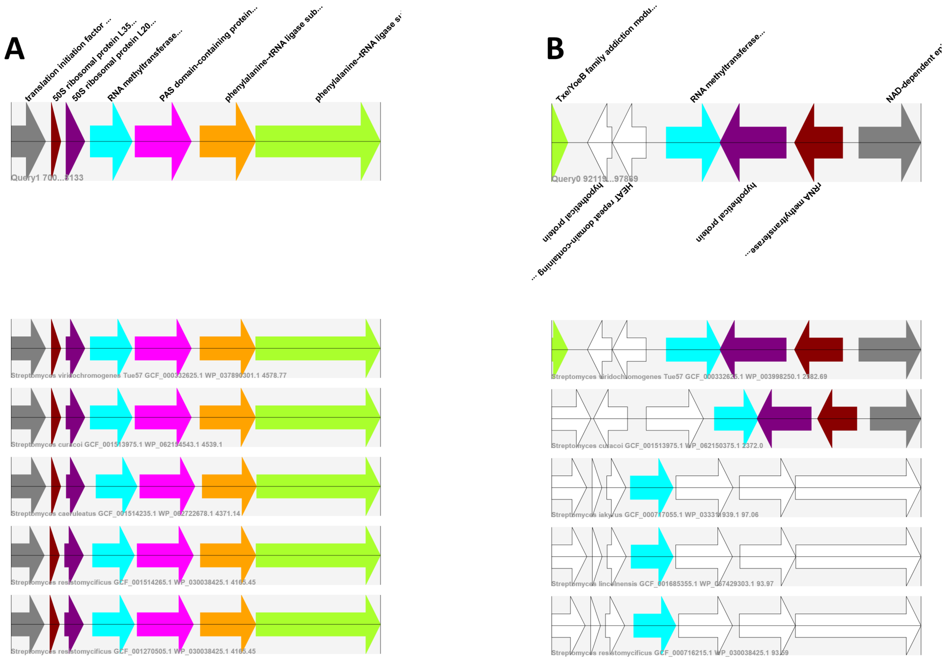

Figure S1. SYN-view result of *Streptomyces viridochromogenes* Tue57. The figure shows two alignments of NGIs throughout the closest relatives of *Streptomyces viridochromogenes* Tue57 (Table 1). Note that for a clear comparison, only two NGI alignments are shown, while three were found (Supplementary data). **A**: NGI of rRNA methyltransferase which is regularly observed in close relatives. **B**: The NGI of the resistant rRNA methyltransferase is unique to the antibiotic producing strain and can easily be distinguished. The first resulted NGI is identical since its the NGI from the same organism, found in autoMLST search.

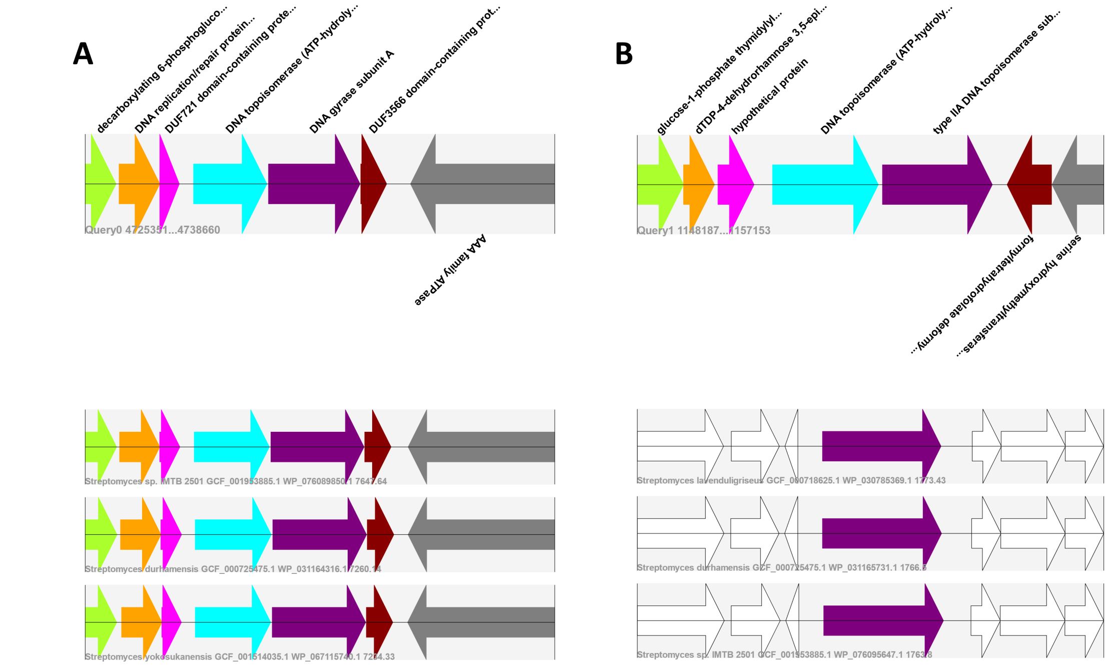

Figure S2. SYN-view result of *Streptomyces roseochromogenes* DS 12.976. Only two of four NGIs are displayed (supplementary data). **A**: NGI of *gyrB* which is regularly observed in close relatives. **B**: The NGI is unique to the strain and can easily be distinguished.

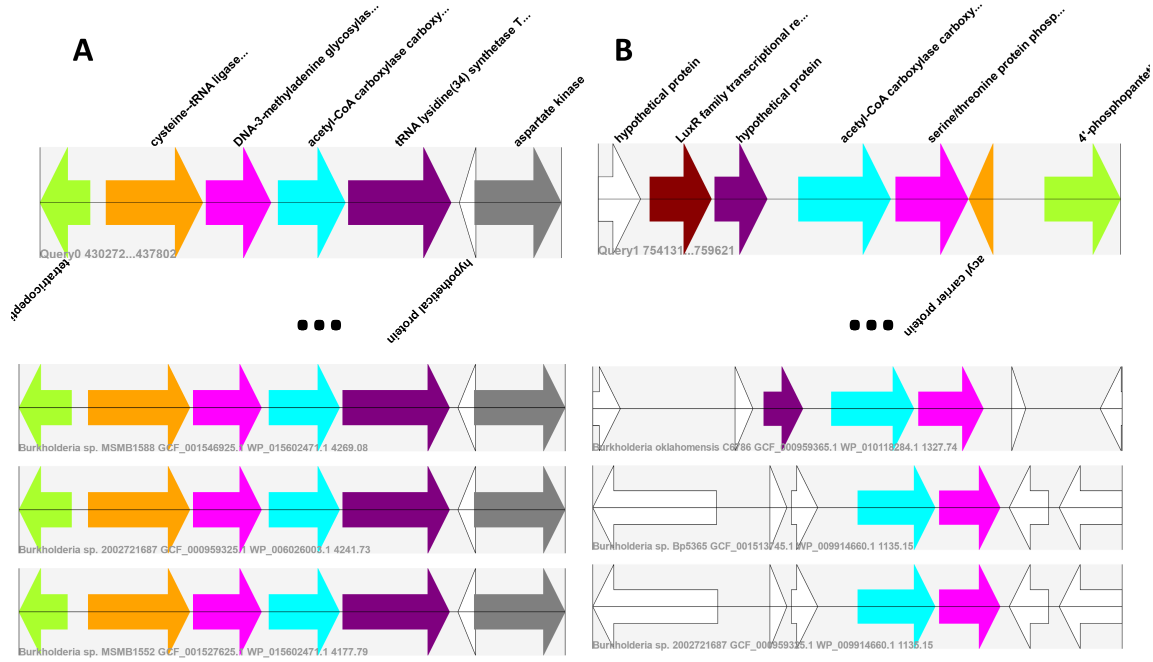

Figure S3. SYN-view result of *Burkholderia thailandensis* E264. Please note that the first displayed NGI of the close relatives is the eleventh NGI in total. All NGIs before are from different *B. thailandensis* strains and show no difference to the query. **A**: NGI of accA which is regularly observed in close relatives. **B**: The NGI is less frequent and differences are observed after evaluation of the results.

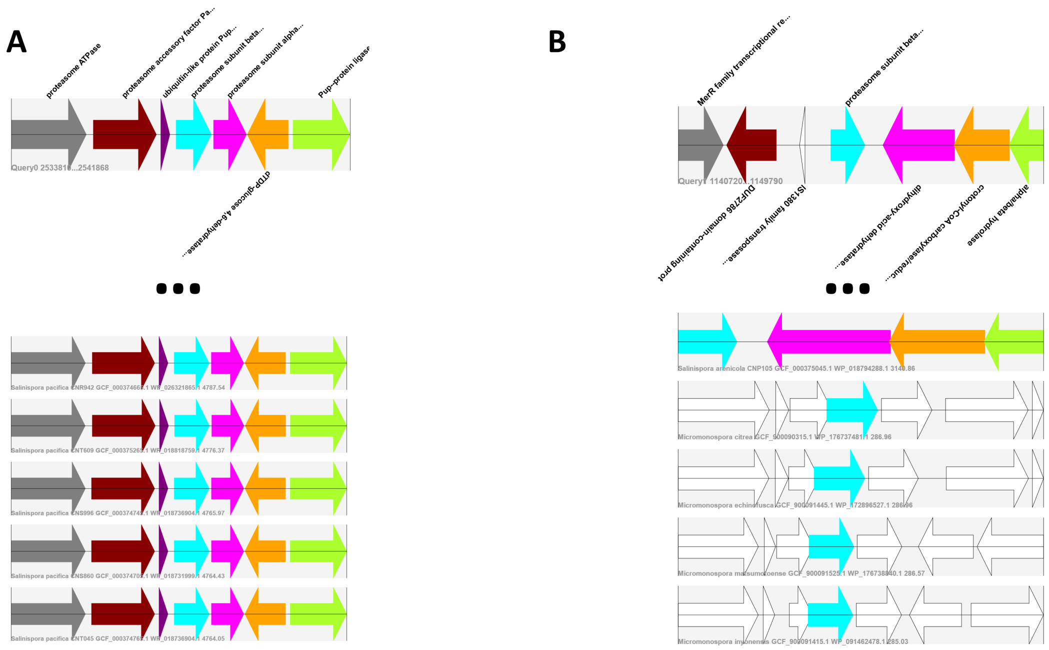

Figure S4. SYN-view result of *Salinospora tropica* CNB-440. Please note that the first displayed NGI of the close relatives is the tenth NGI in total. All NGIs before are from different *S. tropica* strains and show no difference to the query. **A**: NGI of the beta-proteasome subunit which is regularly observed in close relatives. **B**: The NGI is less frequent and differences are observed after evaluation of the results.

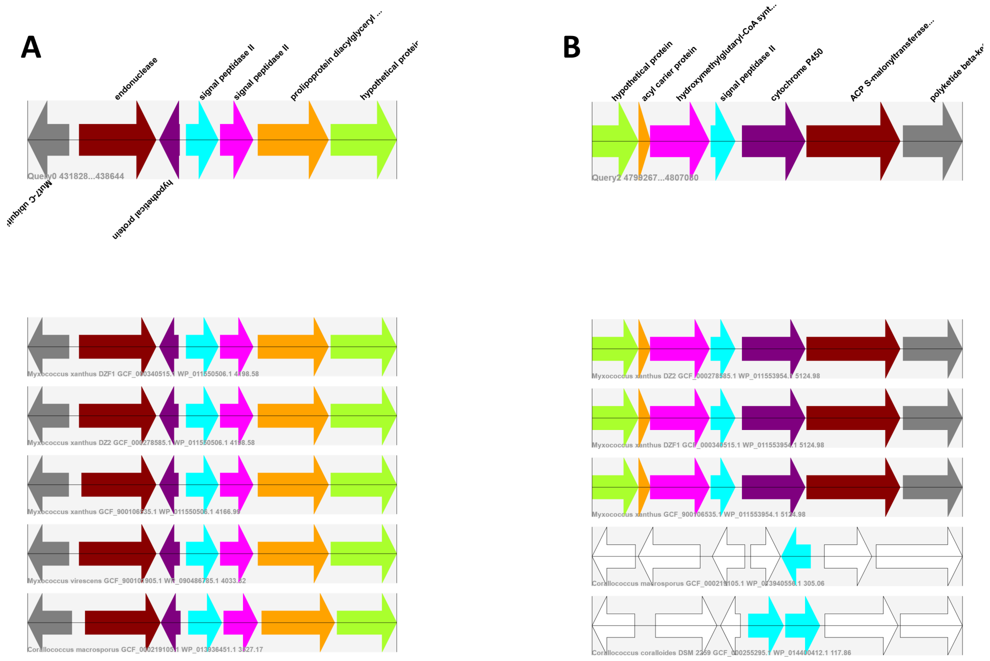

Figure S5. SYN-view result of *Myxococcus xanthus* DK 1622. Only two of three NGIs are displayed (supplementary data). **A**: NGI of the signal peptidase II which is regularly observed in close relatives. **B**: Few *M. xanthus* strains contain the query NGI, but starting at the fouth NGI no similar NGIs can be observed.

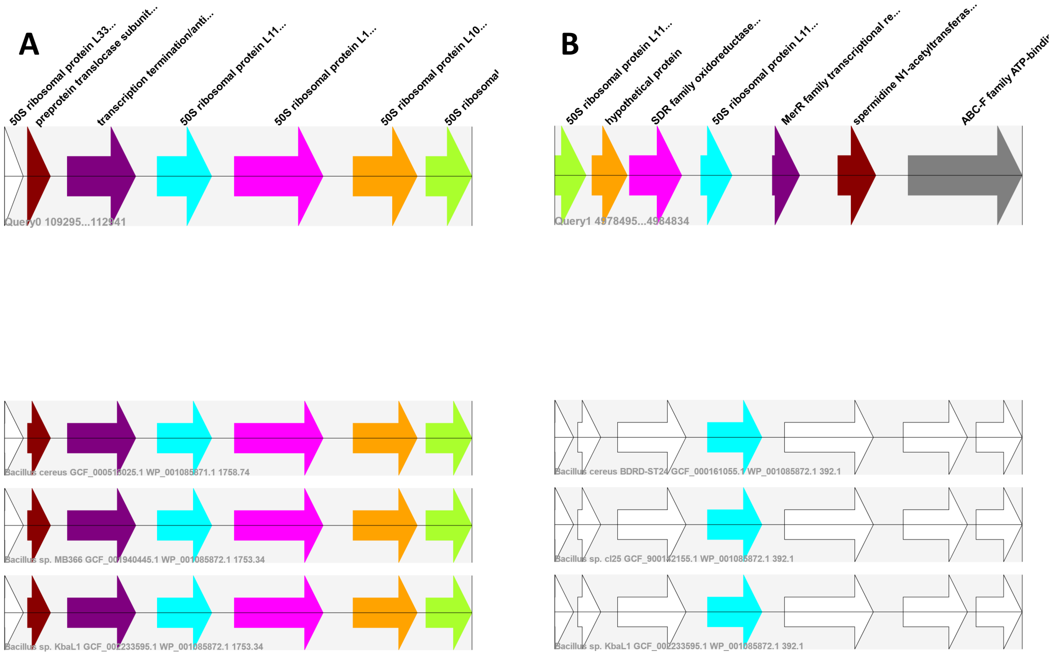

Figure S6. SYN-view result of *Bacillus cereus* ATCC 14579. **A**: NGI of RL11 which is regularly observed in close relatives. **B**: The NGI is unique to the strain and can easily be distinguished.

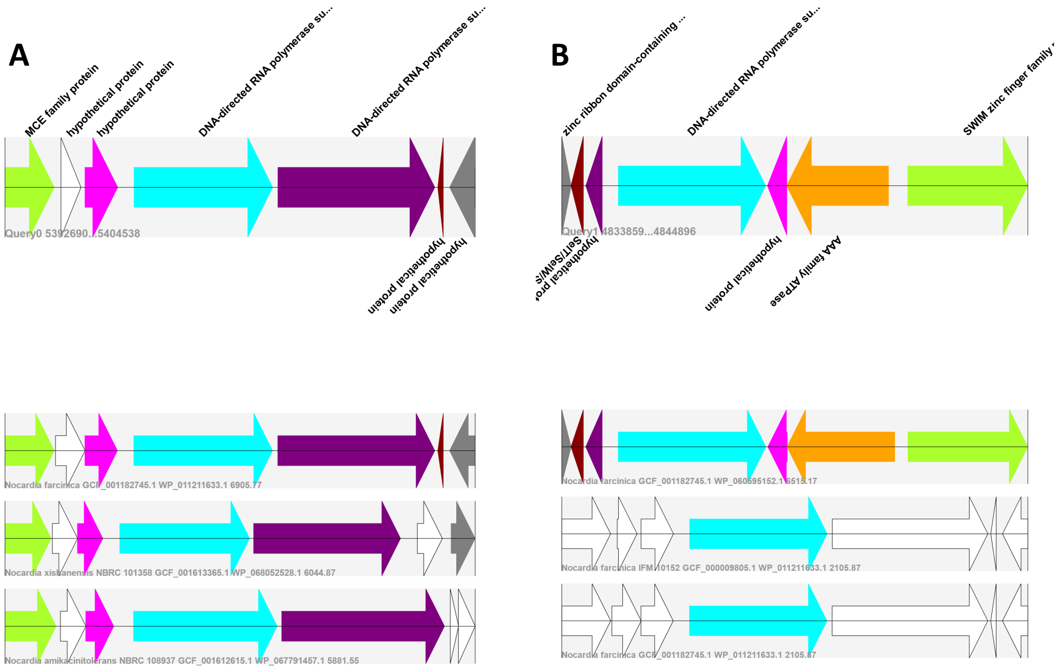

Figure S7. SYN-view result of *Nordica farnica* IFM 10152. **A**: NGI of *rpoB* which is regularly observed in close relatives. **B**: One *N. farnica* strain contains the same NGI. The NGI is unique to all other close relatives.

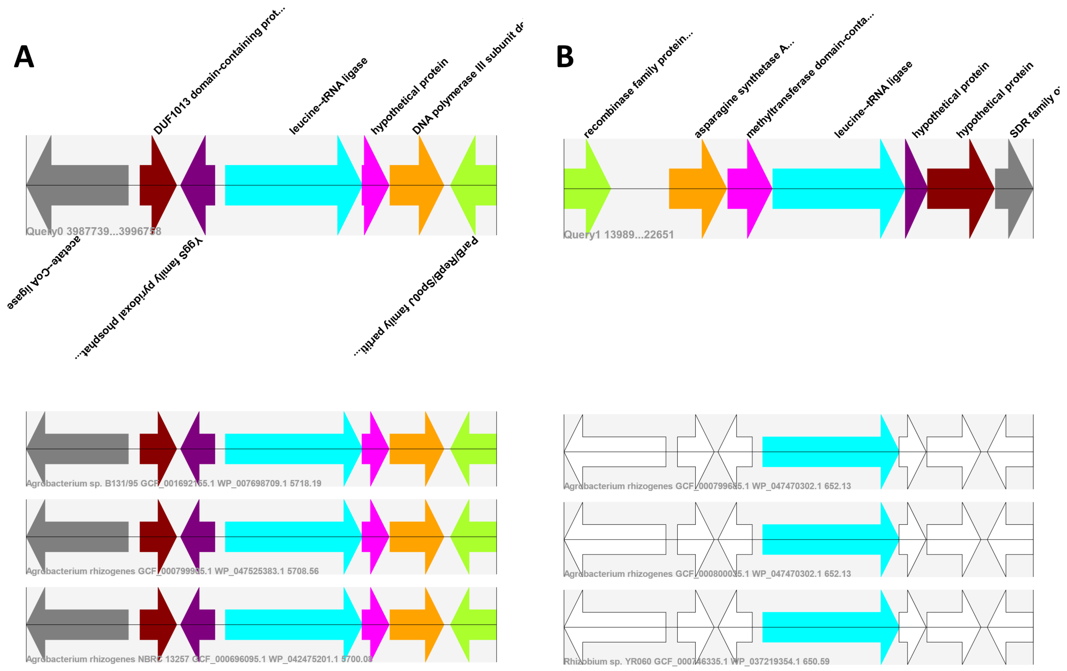

Figure S8. SYN-view result of *Agrobacterium radiobacter* K84. Only two of five NGIs are displayed (supplementary data). **A**: NGI of the Leu-tRNA synthase which is regularly observed in close relatives. **B**: The NGI is unique to the strain and can easily be distinguished.

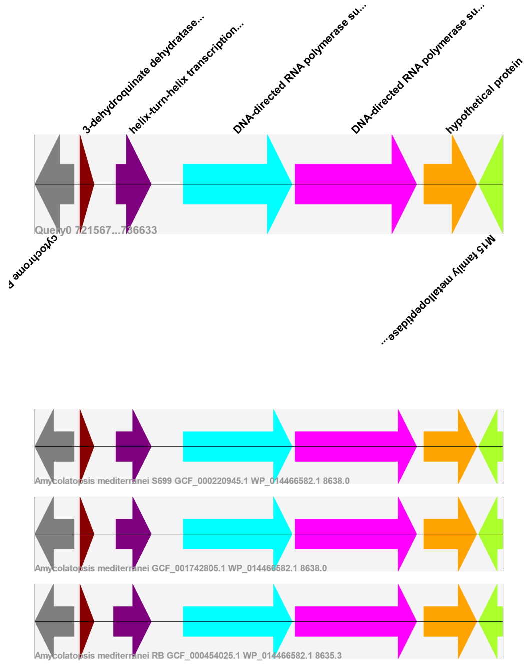

Figure S9. SYN-view result of *Amycolatopsis mediterranei* S699. The NGI of *rpoB* does not show differences of close relatives
